# Exploring *Trichoderma* Species in Industrial Wastewater: Morphological and Molecular Insights from Isolates

**DOI:** 10.3390/life14060750

**Published:** 2024-06-12

**Authors:** Syeda Bint-e-Zahira, Abdul Nasir Khalid, Nousheen Yousaf, Muhammad Iqbal, Tauseef Anwar, Huma Qureshi, Saleh H. Salmen, Mohammad Javed Ansari

**Affiliations:** 1 Institute of Botany, University of the Punjab, Lahore 54590, Pakistan; 2 Department of Botany, Government College University, Lahore 54000, Pakistan; 3 Department of Botany, University of Chakwal, Chakwal 48800, Pakistan; 4 Department of Botany, The Islamia University of Bahawalpur, Bahawalpur 63100, Pakistan; 5 Department of Botany and Microbiology, College of Science, King Saud University, Riyadh 11451, Saudi Arabia; ssalmen@ksu.edu.sa; 6 Department of Botany, Hindu College Moradabad, Mahatma Jyotiba Phule Rohilkhand University Bareilly, Bareilly 244001, India; mjavedansari@gmail.com

**Keywords:** biodiversity, industrial ecology, environmental microbiology, bisorptional activity, ecological roles

## Abstract

The genus *Trichoderma* holds economic significance due to its widespread distribution and diverse applications, including biological control, enzyme production, and various biotechnological uses. The accurate identification of *Trichoderma* species is crucial given their close association with human activities. Despite previous efforts in classification, a comprehensive analysis combining morphological and molecular approaches is necessary. This study focuses on the isolation of four *Trichoderma* species from industrial wastewater in Pakistan, expanding on the known diversity in the region; isolation involved collecting samples from industrial wastewater effluents at specific sites in Punjab, Pakistan. *Trichoderma* strains were cultured and purified on solid media, with subsequent biomass production for bisorptional activity. Morphological characterization included colony features and microscopic examinations. DNA extraction, polymerase chain reaction (PCR), and sequencing of the internal transcribed spacer (ITS) region were conducted for molecular analysis. Phylogenetic analysis was performed using the Maximum Likelihood Algorithm. The study identified three *Trichoderma* species, viz. *T. citrinoviride*, *T. erinaceum*, and *T. longibrachiatum*. Each species was characterized morphologically and supported by molecular–phylogenetic analysis. Illustrations of microscopic features and a phylogenetic tree based on the ITS-nrDNA region were recorded. *T. citrinoviride* and *T. longibrachiatum*, isolated from steel mill and tanneries wastewater, respectively, were differentiated based on morphological characteristics such as phialides and conidia. The combination of morphological and molecular techniques enhances the accuracy of species identification. The study highlights the significance of *Trichoderma* in industrial wastewater environments and underscores the need for continued research in this area. Future research should focus on exploring the ecological roles and potential applications of the newly identified *Trichoderma* species. Additionally, further investigations into the biotechnological potential of these species, including enzyme production and bioremediation capabilities, would contribute to their practical applications.

## 1. Introduction

In recent years, the treatment of industrial wastewater has become a critical environmental concern due to its potential adverse effects on ecosystems and human health [1]. Traditional methods of wastewater treatment often prove insufficient, prompting the exploration of alternative, sustainable solutions. Among these, the utilization of microorganisms holds significant promise [2]. *Trichoderma* species, renowned for their diverse metabolic capabilities and robust enzymatic machinery, have emerged as compelling candidates for bioremediation applications. Their proficiency in degrading a wide range of organic pollutants, coupled with their ability to thrive in varied environmental conditions, renders them particularly attractive for the treatment of industrial effluents [3].

The genus *Trichoderma* presents an intriguing focus within the field of mycology, boasting a diverse array of species with varied ecological roles and economic significance. Originally introduced by Persoon [1] and subsequently monographed by Rifai [2], the genus has since been the subject of in-depth analyses by Bissett [3,4,5,6,7] and Samuels et al. [8], who provided comprehensive insights into the morphological variations within the genus. The economic importance of *Trichoderma* spans various domains, from its role as a biological control agent to the production of enzymes with agricultural and biotechnological applications [9,10,11]. *Trichoderma* species are well known for their robust biotechnological applications, particularly in the context of environmental bioremediation. Recent studies have demonstrated the efficacy of *Trichoderma harzianum* in removing recalcitrant dyes, such as crystal violet (CV), from industrial wastewater [12]. Similarly, in a dual-chamber fungal microbial fuel cell (FMFC), a mixed culture of *T. harzianum* and *Pseudomonas fluorescens* achieved the complete removal of acetaminophen (APAP) and its by-product 4-aminophenol (PAP) within approximately 7 h [13].

*Trichoderma* species inhabit a myriad of environments, including soil, marshes, water bodies, and decaying woods [14,15,16]. Their intimate association with diverse habitats emphasizes the need for accurate identification, especially considering the impact of human activities on these fungal communities [17]. While morphological identification methods laid the foundation for *Trichoderma* taxonomy, molecular techniques have become indispensable for ensuring precision and reliability in species delineation.

In the particular domain of industrial wastewater, the investigation into *Trichoderma* takes on increased importance. Industrial activities introduce a myriad of pollutants into water bodies, creating unique ecological niches that demand adaptation from resident microorganisms. *Trichoderma*, known for its adaptability and diverse metabolic capabilities, becomes a compelling subject for investigation in these environments. The potential ecological roles, enzymatic activities, and biotechnological applications of *Trichoderma* species in industrial wastewater emphasize the importance of this study [18,19]. Despite the existing body of knowledge on *Trichoderma*, certain gaps persist, particularly in understanding its diversity within industrial settings [20]. This study bridges the existing gap by reporting three *Trichoderma* species isolated for the first time from industrial wastewater in Pakistan. The incorporation of both morphological and molecular techniques provides a holistic approach to species identification, ensuring the accuracy and reliability of the reported findings. The rationale behind this exploration lies in the unique challenges posed by industrial wastewater environments and the potential of *Trichoderma* species to adapt and thrive in these conditions. The morphological characterization offers a tangible understanding of the visible traits of the isolated species, while molecular–phylogenetic analysis delves into the genetic underpinnings, enhancing the precision of species identification. Moreover, the study contributes to the broader understanding of fungal diversity in industrial contexts, shedding light on the ecological resilience and adaptive strategies of *Trichoderma*.

The research gap lies in the need for a deeper understanding of *Trichoderma* species within industrial wastewater environments. While there is acknowledgment of the genus Trichoderma’s ecological roles and economic importance, there is a specific emphasis on the scarcity of knowledge regarding their diversity and behavior within industrial settings. This lack of comprehensive understanding creates a gap in the literature, highlighting the necessity for further investigation. The aim of this study is to investigate and characterize Trichoderma species obtained from industrial wastewater, employing both morphological and molecular analyses. Morphological characterization involves examining colony morphology, microscopic features, and other relevant traits. Molecular techniques, including DNA extraction, PCR, and sequencing, are utilized to analyze the internal transcribed spacer (ITS) region of fungal nuclear ribosomal DNA (nrDNA). Additionally, a phylogenetic tree is constructed based on the ITS-nrDNA sequences to elucidate the evolutionary relationships among the isolated Trichoderma species. The study also reports and discusses the findings, focusing on the identification of Trichoderma species and their potential ecological roles in industrial wastewater environments.

## 2. Materials and Methods

### 2.1. Sampling Sites, Strain Culture, and Biomass Production

In the present study, *Trichoderma* strains were isolated from industrial wastewater effluents sourced from three specific locations in Lahore, Punjab, Pakistan: Steel Mill Sheikhupura Road, Hafeez Shafih Tanneries Ferozepur Road, and Nishat Textile Mill Ferozepur Road. These sites were chosen for their representation of diverse industrial activities, each contributing distinct pollutants to the wastewater, aligning with recent research emphasizing the importance of considering specific industrial contexts when studying microbial communities in wastewater. Wastewater samples were collected in sterile containers and immediately stored at 4 °C to preserve the integrity of the fungal isolates for subsequent isolation procedures.

To isolate *Trichoderma* strains, 100 µL of collected polluted water from each sample was evenly spread on a solidified medium in Petri plates containing 2% Potato Dextrose Agar (PDA). Control plates without water inoculation were also prepared to discern the impact of pollutants on fungal growth. Twenty replicates were established for each wastewater sample to ensure a robust experimental design following the latest protocols [17]. As fungal colonies emerged on the Petri plates, a series of purification steps were undertaken. Emerging colonies were transferred to freshly prepared PDA plates using sterile techniques. This sub-culturing process was performed in replicates of five to maintain the purity of the cultures. All fresh cultures were incubated at 20 °C, a temperature optimized based on recent studies indicating it as the optimal growth condition for Trichoderma [18]. For biomass production, pure cultures were repeatedly cultured on a 2% PDA medium. This repeated culturing ensured sufficient biomass for subsequent studies and was crucial for exploring the biorational activity of the isolates [19].

### 2.2. Isolation and Morphological Characterization

*Trichoderma* strains were further isolated by cultivating them on both 2% PDA and Malt Extract Agar (MEA) media. The use of multiple media types is important for capturing the diverse array of Trichoderma species present in complex environmental samples [20]. Pure cultures of each colony were obtained through successive sub-culturing, ensuring the preservation of distinct morphological traits. The morphological characterization included a comprehensive assessment of colony features, including color, appearance on the medium, texture, growth patterns, aggregation of hyphae, margins, elevation, odor, exudate, and reverse colony characteristics. These detailed observations provide insight into the adaptive features of Trichoderma in response to industrial pollutants [21].

For microscopic analysis, a block of the medium containing a growing fungal colony was longitudinally sliced, and thin sections were mounted in 0.05% Trypan blue in Lactophenol. The slides were then subjected to microscopic analysis, and the final prepared slides were photographed for documentation. This high-resolution imaging approach aligns with recent trends emphasizing its importance for accurate morphological characterization (Recent Advances in High-Resolution Imaging for Fungal Morphology, [2022]). Drawings of microscopic features were generated through camera lucida, ensuring precision in illustrating intricate fungal structures for accurate taxonomic identification [22].

### 2.3. DNA Extraction, PCR, and Sequencing

Genomic DNA extraction from fresh cultures was conducted using the Extract-N-AmpTM (SIGMA, St. Louis, MO, USA) plant kit in accordance with the manufacturer’s instructions [23]. The polymerase chain reaction (PCR) targeted the internal transcribed spacer (ITS1 and ITS2) along with the 5.8 S region of fungal nrDNA. Primers ITS1F, ITS1, and ITS4 were selected following the established protocol [24]. PCR conditions, encompassing denaturation, annealing, and extension, were optimized based on the current literature concerning fungal ITS amplification [25]. The verification of PCR product yield was conducted through agarose gel electrophoresis, a standard procedure in molecular biology, ensuring DNA fragment integrity and adherence to contemporary molecular biology standards [26]. DNA products were subsequently purified and sequenced at Macrogen Inc., South Korea. The selection of this sequencing facility was guided by its reputation for providing high-quality sequencing services, aligning with recent studies that stress the significance of reliable sequencing for accurate phylogenetic analysis [27].

### 2.4. Phylogenetic Analysis

The obtained DNA sequences underwent thorough editing and correction using Bio Edit software v. 7.0.5.3. Sequence alignment was performed using MUSCLE alignment software v. 3.8.31, aligning with the contemporary practice of utilizing advanced bioinformatics tools for precise sequence alignment [28]. The construction of the phylogenetic tree employed the Maximum Likelihood Algorithm, a methodological choice influenced by recent advancements in phylogenetic analysis and its preference for accurate tree reconstruction [29]. MEGA 7 software was utilized for tree construction, with a bootstrap consensus tree inferred from 1000 replicates to ensure the robustness of the analysis. Bootstrap values exceeding 50% were included in the tree, in accordance with standard practices in phylogenetic studies [30]. All generated ITS-nrDNA sequences of Trichoderma species were responsibly deposited in GenBank, ensuring the availability of this valuable genetic information for the scientific community.

## 3. Results and Discussion

The isolation and characterization of fungal taxa from industrial wastewater effluents yielded valuable insights into the mycobiota present in these complex environments. A comprehensive analysis of the isolated fungi revealed a diverse array of fungal species inhabiting the sampled sites. Overall, the genus *Trichoderma* was found to be prevalent across all sampled locations, indicating its widespread occurrence in industrial wastewater. This high frequency emphasizes the ecological significance of *Trichoderma* species in these industrial settings and highlights their potential role in bioremediation processes. In addition to Trichoderma, other fungal genera such as *Aspergillus*, *Penicillium*, and *Fusarium* were also identified in the sampled water sources, albeit at lower frequencies. These findings suggest a complex fungal community composition within industrial wastewater, with multiple genera coexisting and potentially interacting with each other and the surrounding environment.

The occurrence of *Trichoderma* species across diverse industrial activities, including steel manufacturing, tanneries, and textile mills, further emphasizes their adaptability to varying pollutant types and concentrations. This adaptability may be attributed to the versatile metabolic capabilities and enzymatic machinery possessed by *Trichoderma* spp., allowing them to thrive in challenging environmental conditions. Understanding the distribution and abundance of *Trichoderma* and other fungal taxa in industrial wastewater is crucial for assessing the potential impact of these microorganisms on ecosystem dynamics and human health. Furthermore, elucidating the ecological roles and interactions of these fungi can inform the development of targeted bioremediation strategies aimed at mitigating the adverse effects of industrial pollution on the environment. By providing a comprehensive overview of the isolated fungi, including the occurrence and frequency of Trichoderma species, this study enhances our understanding of the mycobiota inhabiting industrial wastewater environments and lays the groundwork for future research on fungal ecology and bioremediation. Moving forward, we provide a detailed description of the isolated fungal taxa, focusing on their morphological characteristics and phylogeny.

**Figure 1:**
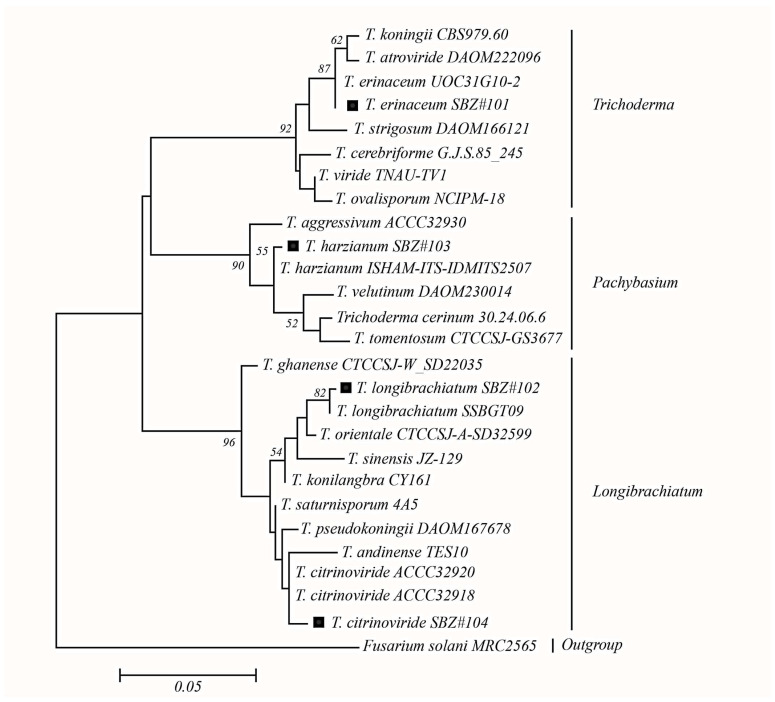
Molecular phylogenetic analysis of *Trichoderma* spp. based on ITS-nrDNA sequences.

***Trichoderma citrinoviride Bissett, Can. J. Bot., 62(5): 926(1984). Figs. 1 & 5 (GenBank Accession no. KJ093620)***

*Trichoderma citrinoviride* Pakistan, isolated from effluents of Steel mills wastewater, Sheikhupura road, Lahore, Punjab, 15 May 2021, SBZ # 104 (GenBank Accession no. KJ093620).

*Colony morphology*: Yellowish to white, irregular, little free aerial mycelium with 3–5 cm in diameter. The margins undulated, and the texture fluffy. Odor was detected. The reverse was off-white.

*Microscopic Characteristics*: Conidiophore comprises a long fertile axis, 1–7 µm wide. Phialides arise singly from the main axis for a short distance, in whorls of a few at the tips of the lateral branches, cylindrical and slightly enlarged and squat, especially when in whorls, 5–15 µm long. Conidia were green, smooth, oblong to ellipsoidal, 2.5–6.5 × 1.5–2.8 µm (Figures S1A and S2A).

*Ecological and Biological Features*: It is a widespread taxon, mostly known to occur in soil, water, wood, and as an endophyte [31,32]. It is a source of several biologically active compounds and acts as an effective biological control agent against many fungal plant pathogens. It can also cause diseases in immunosuppressed humans [33,34].

*BLAST results*: one ITS-nrDNA sequence was generated from Isolate SBZ#104 which was 487 nucleotide long. BLAST results of this sequence (KJ093620) showed 99.39% identity with 13 sequences of *T. citrinoviride* (MN114623, MN547406, MT210518, MN756678, MN242706, MN187551, MG972803, MG972800, MH624144, MH865864, MH861877, MH67131, MK939516) with 100% query coverage. Phylogenetic analysis also supported the similar trend of clustering as our second isolate fitted well with other sequences of *T. citrinoviride* in the phylogram.

***Trichoderma erinaceum Bissett, 2003, Can. J. Bot., 81: 583. Figs. 2 & 6 (GenBank Accession no. KJ093622)***

*Trichoderma erinaceum* Pakistan, isolated from effluents of Nishat Textile Mills, Ferozepur Road, Lahore, Punjab, 15 May 2021, SBZ # 101 (GenBank Accession no. KJ093622).

*Colony morphology*: White hyphae at first, changing to off-white with time, flat lawns in concentric rings with some tendency to form flat pustules becoming slightly convex later. Initially, only white hyphal threads spread concentrically around the original inoculation, converting thick hyphal aggregation to pustules after 15 days, not embedded, some yellow patches appear after 15 days, with no diffusing pigment. The margins were filiform, and the texture was thread-like at the start but was compact cottony at maturity. Odor is more or less like ethylene. Reverse chartreuse to light yellow to off-white.

*Microscopic Characteristics*: The hyphae were hyaline, thick-walled, septate, and 1.8–2.5 µm wide. Conidiophore was hyaline, cylindrical, thick-walled, smooth, branched at different angles, branching paired or not, and 2.0–3.0 µm wide. Phialides were hyaline, cylindrical with almost the same width at the neck and base, solitary and in pairs, straight, and 5.4–8.7 µm long. Conidia was light yellow-green, ellipsoidal, thick-walled, smooth, and 3.9–5.1 × 2.5–3.1 µm wide. Chlamydospores were orange-yellow, globose, and sub-globose, both intercalary and terminal, thick-walled, smooth, and 8.9–12.4 µm (Figures S1B and S2B).

*Ecological and Biological Features*: *T. erinaceum* is an effective biocontrol agent against plant pathogenic and soil-borne fungi [35]. It is also known for the production of enzymes such as protease, chitinase, and β-1,3-glucanase [36].

*BLAST results*: ITS-nrDNA sequence of SBZ # 101 was 458 nucleotide long and when BLAST-searched in GenBank, showed 98.7% similarity and 100% query coverage with 12 other sequences of *T. erinaceum* (MT184411, MK990281, MK713516, MK713500, MN173872, MK109827, MK109822, MK109821, MK109820, MK109819, MK109818, MK109816). Our phylogenetic results are also consistent with these BLAST findings.

***Trichoderma harzianum Rifai, Mycol. Pap. 116: 38 (1969). Figs. 3 & 7 (GenBank Accession no. MK530102)***

*Trichoderma harzianum* Pakistan, isolated from effluents of Nishat Textile Mills, Ferozepur Road, Lahore, Punjab, 15 May 2021, SBZ # 103 (GenBank Accession no. MK530102).

*Colony Morphology*: The colony morphology was greenish gray, and zones of different colors were found, at some places gray color dominated, while at others green color dominated, thick white hyphal mass at margins at younger stages which converted into colony color with time, growth was fast, and 9.2 cm in diameter after 5 days of inoculation, circular, flat, cottony to fluffy, dense sporulation and with no distinct odor. The margins were smooth, filiform, and white at younger stages while greenish gray at maturity. The texture was fluffy. The exudate was colorless. The odor was not distinct. Reverse four distinct zones at the reverse side, central light brown, next off-white, third is gray, and fourth white, smooth appearance at reverse.

*Microscopic Characteristics*: The conidiophore was hyaline, septate, thick-walled, highly branched at right angle, and 2.9–3.4 µm wide. Phialides were flask-shaped, hyaline, base broader, at right angle to the conidiophore, in clusters of three, arise from primary and secondary branches, short and broad, ovoid with a distinct neck, and 4.1–6.5 µm long. Conidia was brilliant yellow-green, globose to sub-globose to ellipsoidal, thick-walled, smooth, 3.0–4.0 × 2.8–3.3 µm. Chlamydospores were rare, orange-yellow, thick-walled, smooth, globose, and 5.8–7.9 µm (Figures S1C and S2C).

*Ecological and Biological Roles*: *T. harzianum* is known for its potential as a biocontrol agent against plant pathogenic and soil-borne fungi. Additionally, it may contribute to ecosystem dynamics through its enzymatic activities, including the production of protease, chitinase, and β-1,3-glucanase, which can influence nutrient cycling and organic matter decomposition in wastewater environments.

*BLAST results*: A BLAST search using a 531-nucleotide-long ITS sequence of isolate SBZ # 103 showed a 100% match by identity and query coverage with many other sequences reported in GenBank. These included four sequences of *Trichoderma* sp. (MT250832, MT250830, MK871264, MK285188), one of T. aff. harzianum (KP009253), two sequences of *T. harzianum* (MW689515, AF483587), etc. Phylogenetic analysis assisted in accurate identification of our isolate as *T. harzianum*.

***Trichoderma longibrachiatum Rifai, 1969, Mycological Papers 116: 42. Figs. 4 & 8 (GenBank Accession no. KJ093619)***

*Trichoderma longibrachiatum* Pakistan, isolated from effluents of Hafeez Shafih Tanneries, Ferozepur Road, Lahore, Punjab, 15 May 2021, SBZ # 102, (GenBank Accession no. KJ093619).

*Colony Morphology*: The colony morphology was middle green in the center surrounded by white hyphal mass, flat, wooly becoming wet and compact in time, 6.5 cm in diameter after 5 days of inoculation, hyphal and conidial tufts, conidial mass dark green, sometimes mottled with white flecks and often with inconspicuous wefts of yellow hyphae, and yellow pigment diffusing through the agar. Margins are undulated at a young stage and become undefined at maturity. The texture was powdery. The odor was not distinct. Reverse yellow color due to the exude of the colony.

*Microscopic Characteristics*: Hyphae was hyaline, smooth, branched, thick-walled, and 3.1–6.7 µm wide. Conidiophore was hyaline, thick-walled, smooth, septate, branched at different angles, branching more on one side, long side branches bearing phialide, tapering at the end, and 3.5–4.2 µm wide. Phialides were hyaline, cylindrical to flask-shaped, constricted at the base, attached to the conidiophores at right angles on long branches, intercalary common, opposite, solitary mostly, arise at irregular intervals, curved to some extent, endings not well defined, and 3–13 µm long. Conidia was unicellular, light green, sub-globose, thick-walled, smooth, and 3.0–5.8 × 2.0–3.3 µm (Figures S1D and S2D).

*Ecological and Biological Features*: *T. longibrachiatum* occurs in soil and as an endophyte. It plays a role in bioremediation [37], as a biological control agent [38], and in the production of enzymes and metabolites [39,40,41,42,43]. This taxon is considered an emerging pathogen in humans with weak immune systems [44].

*BLAST results*: A sequence similarity search using BLAST of the ITS sequence (499 nucleotide long) of isolate SBZ # 102 revealed a high level of sequence similarity with *T. longibrachiatum* (MG815134), i.e., 99.8%. Our sequence (MK530103) also showed 97.84% identity with more than 100 sequences of *T. longibrachiatum* submitted in GenBank (such as PP620789, PP538036, PP389586, OQ511502, PP336466, PP217389, OR551447, etc.). The phylogenetic findings confirmed the identity of strain SBZ#102 as *T. longibrachiatum*.

### Phylogenetic Analysis

The phylogenetic analysis of fungal isolates was carried out using the Maximum Likelihood Method based on the Kimura 2-parameter model [45]. The tree with the highest log likelihood (−2149.9432) is shown (Figure 1). Table 1 presents a list of taxa used in phylogenetic analyses along with their respective GenBank accession numbers.

A discrete Gamma distribution was used to model evolutionary rate differences among sites [(5 categories (+G, parameter = 0.2517)]. The sequences included in this analysis comprise 611 characters, out of which 81 were parsimony-informative sites, 435 were conserved, and 162 characters were variable. The phylogram consists of three major clades, i.e., *Longibrachiatum*, *Pachybasium*, and *Trichoderma*. Clades were named based on a section of the genus Trichoderma [46]. Phylogenetic analysis has confirmed the identity and placement of *Trichoderma* species, which are presented here as new records.

This study contributes valuable insights into the species diversity of *Trichoderma* in Pakistan, particularly in the context of industrial wastewater environments. The combination of morphological and molecular analyses enhances our understanding of these fungi’s ecological roles and potential applications in biotechnology, agriculture, and environmental management. During the present investigation, four isolates of *Trichoderma* were identified as *T. citrinoviride*, *T. erinacium*, *T. longibrachiatum*, and *T. harzianum* from industrial effluents. Out of these, the first three taxa are described here as new records from Pakistan based on morphological and molecular phylogenetic analysis. *T. citrinoviride* and *T. longibrachiatum* were isolated from steel mill wastewater and tanneries wastewater, respectively, belonging to the *Trichoderma* Sect. *Longibrachiatum*. These two species can be differentiated due to some morphological characteristics; for example, phialides in *T. citrinoviride* are in whorls, broader at the base and narrower at the tip, and tend to be cylindrical, while in *T. longibrachiatum*, the phialides are straight and arise singly. Conidia in *T. citrinoviridi* are oblong to ellipsoidal, while in *T. longibrachiatum*, they are sub-globose. The *T. erinaceum* isolated from textile wastewater resembles *T. koningii* in *Trichoderma* [47,48,49]. Both species have smooth walls and ellipsoid–cylindrical conidia. However, conidiophore in *T. erinaceum* has sterile and straight conidiophore extensions, imparting a special spiny appearance to conidiogenous hyphal pustules in fresh colonies. Another different feature is ampulliform phialides in *T. erinaceum* while lageniform in *T. koningii* [50,51,52,53,54,55].

## 4. Conclusions

In this study, the investigation of *Trichoderma* species isolated from industrial wastewater effluents in Pakistan has unveiled valuable insights into the biodiversity of this economically significant genus. The research identified and characterized four species—*T. citrinoviride*, *T. erinaceum*, *T. harzianum*, and *T. longibrachiatum*—using a combination of morphological and molecular techniques. Distinctive features of each species, including colony morphology, texture, odor, and microscopic characteristics, were revealed through morphological analysis. Notably, *T. citrinoviride* and *T. longibrachiatum*, isolated from steel mills and tanneries wastewater, were differentiated based on specific morphological traits. Molecular methods, including DNA extraction, PCR amplification, and sequencing of the ITS-nrDNA region, facilitated a robust phylogenetic analysis, confirming the identity and placement of the *Trichoderma* species. Sequences obtained during the research have been deposited in GenBank for future reference. This study significantly contributes to our understanding of *Trichoderma* diversity in Pakistan, introducing three new records to the previously reported nine species. Beyond taxonomic considerations, the findings have implications for various applications, such as biological control, enzyme production, and bioremediation. Accurate species identification is crucial for harnessing the potential of *Trichoderma* in agricultural and biotechnological contexts. The integration of both morphological and molecular approaches is emphasized, considering the economic and ecological significance of *Trichoderma* in diverse environments. Overall, this research enhances our knowledge of *Trichoderma* species in industrial wastewater ecosystems, opening avenues for future studies on their ecological roles, functional diversity, and potential biotechnological applications.

## Figures and Tables

**Table 1:** A list of taxa used in phylogenetic analyses along with GenBank accession numbers.

Sr. No.	Taxa	Strain/Isolate ID	GenBank Accession No. (ITS)	Country
1.	*Trichoderma aggressivum*	ACCC32930	MF952664.1	China
2.	*Trichoderma andinense*	TES10	MH549084	-
3.	*Trichoderma atroviride*	DAOM 222096	AF456920	USA
4.	*Trichoderma cerebriforme*	G.J.S. 85-245	KP1022	Indonesia
5.	*Trichoderma cerinum*	30.24.06.6	KP009285	Poland
6.	*Trichoderma citrinoviride*	SBZ#104	KJ093620	Pakistan
7.	*Trichoderma citrinoviride*	ACCC32920	MF871574	China
8.	*Trichoderma citrinoviride*	ACCC32918	MF871572	China
9.	*Trichoderma erinaceum*	UOC 31G10-2	KJ381061	Srilanka
10.	*Trichoderma erinaceum*	SBZ#101	KJ093622	Pakistan
11.	*Trichoderma ghanense*	isolate CTCCSJ-W-SD22035	MF383137	China
12.	*Trichoderma harzianum*	SBZ#103	MK530102	Pakistan
13.	*Trichoderma harzianum*	ISHAM-ITS_ID MITS2507	EF568084	-
14.	*Trichoderma koningii*	CBS979.70	AF359262	-
15.	*Trichoderma konilangbra*	CY161	HQ607999	USA
16.	*Trichoderma longibrachiatum*	SBZ#102	MK530103	Pakistan
17.	*Trichoderma longibrachiatum*	SSBGT09	MG815134	-
18.	*Trichoderma orientale*	CTCCSJ-A-SD32599	MF632112	China
19.	*Trichoderma ovalisporum*	NCIPM-18	KU904460	India
20.	*Trichoderma saturnisporum*	4A5	MF379643	Brazil
21.	*Trichoderma pseudokoningii*	DAOM 167678	EU280097	Colombia
22.	*Trichoderma sinensis*	JZ-129	HQ637329	China
23.	*Trichoderma strigosum*	DAOM 166121	DQ083027	USA
24.	*Trichoderma tomentosum*	CTCCSJ-A-GS3677	MF632116	China
25.	*Trichoderma velutinum*	DAOM 230014	DQ083010	Nepal
26.	*Trichoderma viride*	TNAU-TV1	DQ442274	India
27.	*Fusarium solani*	MRC 2565	MH582400	-

## Data Availability

All amplicon sequences and genome sequences have been deposited to GenBank (NCBI). https://www.ncbi.nlm.nih.gov/nuccore/KJ093620. https://www.ncbi.nlm.nih.gov/nuccore/KJ093622. https://www.ncbi.nlm.nih.gov/nuccore/MK530102. https://www.ncbi.nlm.nih.gov/nuccore/KJ093619 (accessed on 26 April 2024).

## Supplementary Materials

The following supporting information can be downloaded at: https://www.mdpi.com/article/10.3390/life14060750/s1, Figure S1: Colony morphology: A. *Trichoderma citrinoviride* B. *Trichoderma. erinaceum* C. *Trichoderma harzianum* D. *Trichoderma longibrachiatum* (Bars 1 cm); Figure S2. Illustrations of micro-morphological characters- A. *Trichoderma citrinoviride* B. *Trichoderma. erinaceum* C. *Trichoderma harzianum* D. *Trichoderma longibrachiatum* (Bars 5 µm); Table S1: ITS-nrDNA sequences of *Trichoderma* submitted in NCBI database.
