# Supplementary material for: Exploring Trichoderma Species in Industrial Wastewater: Morphological and Molecular Insights from Isolates
Source: Life (Basel). 2024 Jun 12;14(6):750. doi: 10.3390/life14060750 (PMC11204433; doi:10.3390/life14060750)
Supplement: Supplementary file 1 [file life-14-00750-s001.zip › life-3007494-supplementary.pdf]

Supplementary file

Table S1. ITS-nrDNA sequences of *Trichoderma* submitted in NCBI database

Accession No	Nucleotide sequence
>KJ093620.1	*Trichoderma citrinoviride* internal transcribed spacer 1, partial sequence; 5.8S ribosomal RNA gene, complete sequence; and internal transcribed spacer 2, partial sequence<br>TGTGAACGTTACCAATCTGTTGCCTCGGCGGGATTCTCTGCCC<br>CGGGCGCGTCGCAGCCCCGGATCCCATGGCCCGCCGGAGGA<br>CCAACTCAAACTCTTTTTCTCTCCGTCGCGGCCTACGTCGCG<br>GCTCGTTTTATTTTTGCTCTGAGCCTTTCTCGGCACCCTAGCGG<br>GCGTCTCGAAAATGAATCAAAACTTTCAACAACGGATCTCTT<br>GGTTCTGGCATCGATGAAGAACGCAGCGAAATGCGATAAGTA<br>ATGTGAATTGCAGAATTCAGTGAATCATCGAATCTTTGAACGC<br>ACATTGCGCCCGCCAGTATTCTGGCGGGCATGCCTGTCCGAGC<br>GTCATTTCAACCCTCGAACCCCTCCGGGGGGTCGGCGTTGGGG<br>ATCGGCCCCTCACCGGGCCGCCCCCGAAATACAGTGGCGGTC<br>TCGCCGCAGCCTCTCCTGCGCAGTAGTTTGCACACTCGCACCG<br>GGAGCGCGGCGCGGCCA
>KJ093622.1	*Trichoderma erinaceum* internal transcribed spacer 1, partial sequence; 5.8S ribosomal RNA gene, complete sequence; and internal transcribed spacer 2, partial sequence<br>TGTGAACCATACCAAACTGTTGCCTCGGCGGGTCACGCCCC<br>GGGTGCGTCGCAGCCCCGGAACCAGGCGCCCGCCGGAGGGA<br>CCAACCAAACTCTTTACTGTAGTCCCCTCGCGGACGTTATTTC<br>TTACAGCTCTGAGCAAAAAATTCAAAATGAATCAAAACTTTC<br>AACAACGGATCTCTTGGTTCTGGCATCGATGAAGAACGCAGC<br>GAAATGCGATAAGTAATGTGAATTGCAGAATTCAGTGAATCA<br>TCGAATCTTTGAACGCACATTGCGCCCGCCAGTATTCTGGCGG<br>GCATGCCTGTCCGAGCGTCATTTCAACCCTCGAACCCCTCCGG<br>GGGGTCGGCGTTGGGGATCGGAACAGACGGATCCCGGCC<br>CCGAAATACAGTGGCGGTCTCGCCGCAGCCTCTCCTGCGAGT<br>AGTTTGCACAACTCGCACCGGGAGCGCGGCGCGTCCA
>MK530102.1	*Trichoderma harzianum* isolates SBZ#103 internal transcribed spacer 1, partial sequence; 5.8S ribosomal RNA gene and internal transcribed spacer 2, complete sequence; and large subunit ribosomal RNA gene, partial sequence<br>TGTGAACGTTACCAAACTGTTGCCTCGGCGGGATCTCTGCCCC<br>GGGTGCGTCGCAGCCCCGGACCAAGGCGCCCGCCGGAGGAC<br>CAACCTAAAACTCTTATTGTATACCCCCTCGCGGGTTTTTTTA

	TAATCTGAGCCTTTCTCGGCGCCTCTCGTAGGCGTTTCGAAAA<br>TGAATCAAAACTTTCAACAACGGATCTCTTGGTTCTGGCATCG<br>ATGAAGAACGCAGCGAAATGCGATAAGTAATGTGAATTGCA<br>GAATTCAGTGAATCATCGAATCTTTGAACGCACATTGCGCCCG<br>CCAGTATTCTGGCGGGCATGCCTGTCCGAGCGTCATTTCAACC<br>CTCGAACCCCTCCGGGGGGTCGGCGTTGGGGATCGGCCCTCC<br>CTTAGCGGGTGGCCGTCTCCGAAATACAGTGGCGGTCTCGCC<br>GCAGCCTCTCCTGCGCAGTAGTTTGCACACTCGCATCGGGAGC<br>GCGGCGCGTCCACAGCCGTTAAACACCCAACTTCTGAAATGT<br>TGACCTCGGATCAGGTAGGAA
>KJ093619.1	*Trichoderma longibrachiatum* internal transcribed spacer 1, partial sequence; 5.8S ribosomal RNA gene, complete sequence; and internal transcribed spacer 2, partial sequence<br>TGTGAACGTTACCAATCTGTTGCCTCGGCGGGATTCTCTTGCC<br>CCGGGCGCGTCGCAGCCCCGGATCCCATGGCGCCCGCCGGAG<br>GACCAACTCCAAACTCTTTTTTTCTCTCCCGTCGCGGCTCTGTT<br>TTATTTTTGCTCTGAGCCTTTCTCGGCGACCCTAGCGGGCGTCT<br>CGAAAATGAATCAAAACTTTCAACAACGGATCTCTTGGTTCTG<br>GCATCGATGAAGAACGCAGCGAAATGCGATAAGTAATGTGA<br>ATTGCAGAATTCAGTGAATCATCGAATCTTTGAACGCACATTG<br>CGCCCGCCAGTATTCTGGCGGGCATGCCTGTCCGAGCGTCATT<br>TCAACCCTCGAACCCCTCCGGGGGGTCGGCGTTGGGGATCGG<br>CCCTCACCGGGCCGCCCCCGAAATACAGTGGCGGTCTCGCCG<br>CAGCCTCTCCTGCGAGTAGTTTGCACACTCGCACCGGGAGCG<br>CGGCGCGGCCA

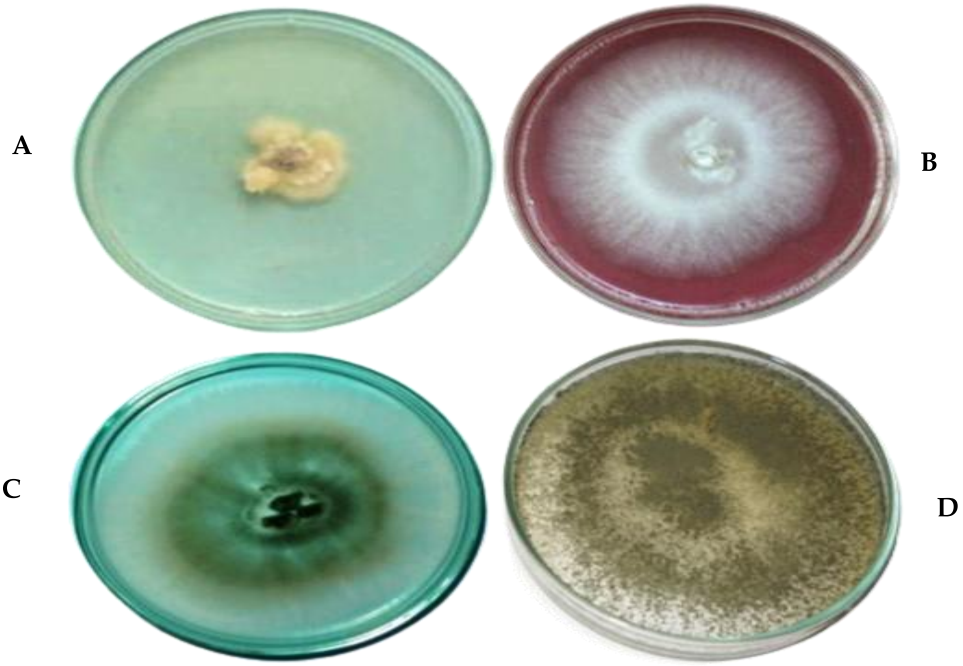

Figure S1. Colony morphology: A. *Trichoderma citrinoviride* B. *Trichoderma. erinaceum* C. *Trichoderma harzianum* D. *Trichoderma longibrachiatum* (Bars 1 cm)

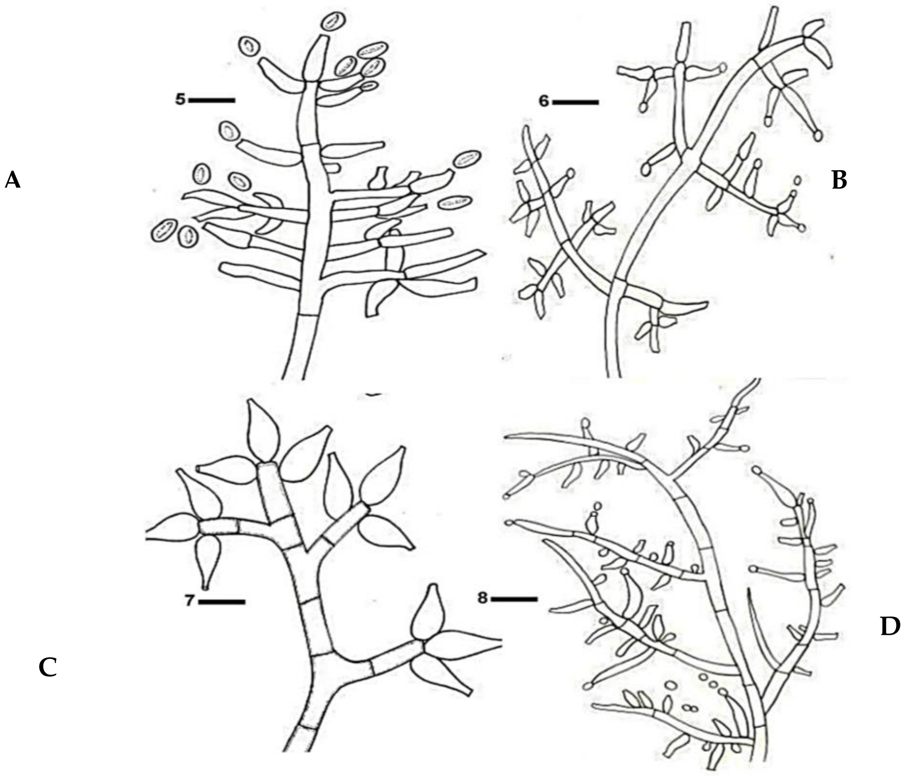

Figure S2. Illustrations of micro-morphological characters- A. *Trichoderma citrinoviride* B. *Trichoderma. erinaceum* C. *Trichoderma harzianum* D. *Trichoderma longibrachiatum* (Bars 5 μm)
